# Laparoscopic transabdominal preperitoneal repair for atypical inguinal hernia associated with adult anorchia: a case report

**DOI:** 10.1093/jscr/rjag273

**Published:** 2026-04-17

**Authors:** Yusuke Yoshida, Ryohei Shoji, Yuki Matsumi, Toshiyoshi Fujiwara

**Affiliations:** Department of Gastroenterological Surgery, Okayama University Graduate School of Medicine, Dentistry and Pharmaceutical Sciences, Okayama, Japan; Department of Gastroenterological Surgery, Okayama University Graduate School of Medicine, Dentistry and Pharmaceutical Sciences, Okayama, Japan; Department of Gastroenterological Surgery, Okayama University Graduate School of Medicine, Dentistry and Pharmaceutical Sciences, Okayama, Japan; Department of Gastroenterological Surgery, Okayama University Graduate School of Medicine, Dentistry and Pharmaceutical Sciences, Okayama, Japan

**Keywords:** adult anorchia, inguinal hernia, laparoscopic transabdominal preperitoneal repair, atypical inguinal anatomy, absent spermatic cord structures

## Abstract

Adult anorchia complicated by inguinal hernia is rare, and its influence on intraoperative anatomy has been insufficiently described. We report a 54-year-old man with left inguinal hernia and congenital absence of testes who underwent laparoscopic transabdominal preperitoneal (TAPP) repair. Preoperative imaging demonstrated a lateral hernia orifice without identifiable spermatic cord structures. Laparoscopic findings revealed absence of normal vas deferens and testicular vessels, resulting in an indistinct internal inguinal ring and atypical inguinal anatomy. Standard anatomical landmarks used for hernia classification and dissection were not applicable, requiring careful preperitoneal dissection with attention to major vessels. TAPP repair enabled comprehensive visualization of the myopectineal orifice and safe mesh placement despite the anomalous anatomy. This case highlights that absence of spermatic cord structures can significantly alter inguinal anatomy and that laparoscopic repair is particularly valuable for both anatomical assessment and safe surgical management in such patients.

## Introduction

Adult anorchia is an extremely rare condition, particularly when diagnosed after puberty [1, 2]. Testicular regression syndrome (TRS), also referred to as vanishing testis syndrome, is thought to result from fetal or perinatal ischemic injury leading to testicular atrophy or disappearance [3]. Although TRS is rare, several reports have suggested a possible association between TRS and inguinal hernia [4]. However, the impact of absent spermatic cord structures on laparoscopic hernia repair and intraoperative anatomical orientation in adulthood has not been sufficiently described. We report a case of adult anorchia complicated by left inguinal hernia in which laparoscopic transabdominal preperitoneal (TAPP) repair enabled direct intraoperative confirmation of atypical hernia anatomy caused by the absence of spermatic cord structures and indistinct internal inguinal ring.

## Case presentation

A 54-year-old man presented with left inguinal bulging and pain. He had undergone bilateral inguinal hernia repair during childhood, at which time bilateral absence of testes within the scrotum had been noted. At 17 years of age, he was evaluated for delayed secondary sexual development and short stature. Endocrinological examination revealed low serum testosterone (0.7 ng/ml) with markedly elevated gonadotropins (luteinizing hormone 78.7 IU/L and follicle-stimulating hormone 101.1 IU/L), consistent with hypergonadotropic hypogonadism. The human chorionic gonadotropin stimulation test was negative, and karyotype analysis demonstrated a normal 46,XY pattern. Laparoscopic exploration was not performed at that time due to the patient’s preference. Based on these findings, bilateral anorchia or severe testicular hypoplasia, suspected to represent testicular regression syndrome, was diagnosed, and hormone replacement therapy was initiated.

**Figure 1:**
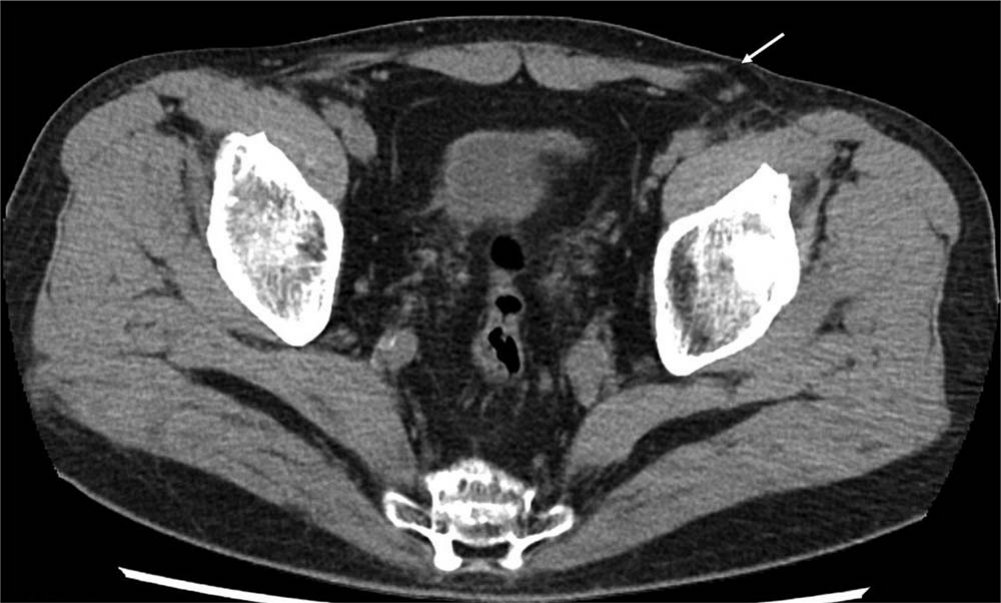
CT findings. The hernial orifice is located lateral to the inferior epigastric vessels (arrow).

At the current presentation, physical examination revealed empty bilateral scrotal sacs and an egg-sized bulge in the left inguinal region. Computed tomography (CT) demonstrated a hernia orifice lateral to the inferior epigastric vessels (Fig. 1); however, neither the vas deferens nor the testicular vessels could be clearly identified, and no testicular tissue was detected in the scrotum, inguinal canal, or abdominal cavity.

**Figure 2:**
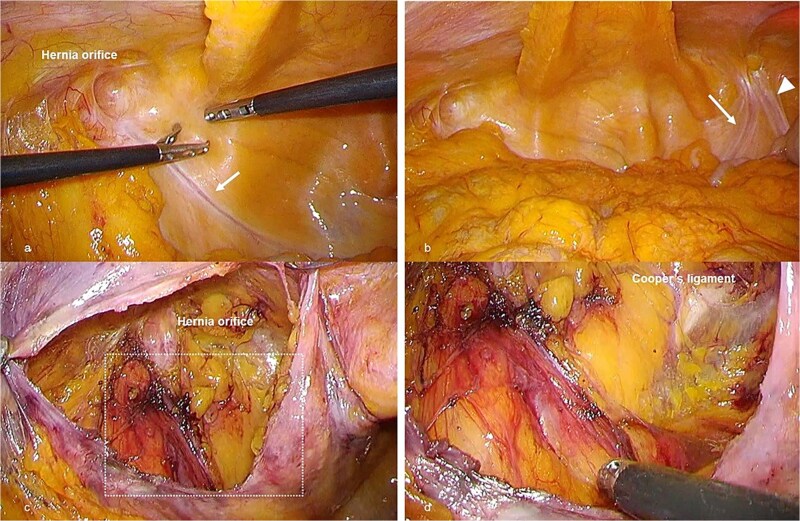
Intraoperative laparoscopic findings during TAPP repair. (a) Laparoscopic view of the left inguinal region before peritoneal incision. No normal spermatic cord structure is visible, but a fibrous structure presumed to be a remnant of the vas deferens is observed immediately beneath the peritoneum (arrow). (b) Contralateral (right) inguinal region viewed through the peritoneum, demonstrating structures presumed to represent remnants of the vas deferens (arrow) and testicular vessels (arrowheads), in contrast to the left side. (c) Preperitoneal view after dissection on the left side. The internal inguinal ring is indistinct, and the absence of definitive spermatic cord structures complicates identification of the dorsal dissection plane. (d) Magnified view of the boxed area in (c), showing no identifiable vas deferens–like structure and no testicular vessels after preperitoneal dissection, as the suspected fibrotic remnant could not be separated from the peritoneum.

Laparoscopic TAPP repair was performed. Intraoperatively, the internal inguinal ring on the left side was indistinct, and no normal spermatic cord structures were present. Testicular vessels were not identified, although a fibrotic, scar-like structure presumed to represent a remnant of the vas deferens was observed immediately beneath the peritoneum. The hernia orifice was located laterally (Fig. 2a). On the right side, remnants of both the vas deferens and testicular vessels were observed beneath the peritoneum (Fig. 2b). Dissection of the presumed vas deferens from the peritoneum was difficult. Careful preperitoneal dissection was performed with particular attention to the external iliac vein, and TAPP repair was successfully completed (Fig. 2c, d). Operative time was 85 minutes with minimal blood loss.

## Discussion

This case represents a rare long-term course of suspected TRS in which absent spermatic cord structures resulted in atypical inguinal anatomy. In such cases, the internal inguinal ring may be poorly defined, making classification of the hernia type difficult. Although the patient had a history of childhood inguinal hernia repair, the atypical anatomy observed in this case was considered to be primarily attributable to congenital absence of spermatic cord structures rather than simple postoperative recurrence. Although the hernia orifice was located laterally and could be interpreted as a recurrent indirect hernia, the absence of spermatic cord structures and the indistinct internal inguinal ring suggested a pathological condition functionally closer to a direct-type hernia caused by abdominal wall weakness.

Conventional inguinal hernia classifications are based on the presence of normal spermatic cord anatomy and the anatomical relationship to the inferior epigastric vessels, and may not adequately describe hernias associated with congenital or developmental anomalies in widely used classifications such as the JHS classification. In the present case, laparoscopic observation allowed comprehensive visualization of the myopectineal orifice and accurate understanding of the atypical anatomy.

In standard TAPP repair, the vas deferens and testicular vessels serve as important landmarks for identifying the appropriate dorsal dissection plane. Their absence may increase the risk of misidentification and vascular injury. Moreover, many open repair techniques assume the presence of a spermatic cord [5], potentially complicating surgery in patients with anomalous anatomy. Laparoscopic repair, by contrast, provides both diagnostic and therapeutic advantages in such settings.

In conclusion, this case highlights that absence of spermatic cord structures can result in atypical inguinal hernia anatomy with an indistinct internal inguinal ring. Laparoscopic TAPP repair is particularly useful in such situations, allowing safe anatomical assessment and effective treatment.

## Data Availability

The data that support the findings of this study are available from the corresponding author upon reasonable request.
